# *In Vivo* Tracking of Cell Therapies for Cardiac Diseases with Nuclear Medicine

**DOI:** 10.1155/2016/3140120

**Published:** 2016-01-12

**Authors:** Mayra Lorena Moreira, Priscylla da Costa Medeiros, Sergio Augusto Lopes de Souza, Bianca Gutfilen, Paulo Henrique Rosado-de-Castro

**Affiliations:** ^1^Departamento de Radiologia, Faculdade de Medicina, Universidade Federal do Rio de Janeiro, 21941-913 Rio de Janeiro, RJ, Brazil; ^2^Instituto de Ciências Biomédicas, Universidade Federal do Rio de Janeiro, 21941-902 Rio de Janeiro, RJ, Brazil

## Abstract

Even though heart diseases are amongst the main causes of mortality and morbidity in the world, existing treatments are limited in restoring cardiac lesions. Cell transplantations, originally developed for the treatment of hematologic ailments, are presently being explored in preclinical and clinical trials for cardiac diseases. Nonetheless, little is known about the possible efficacy and mechanisms for these therapies and they are the center of continuous investigation. In this scenario, noninvasive imaging techniques lead to greater comprehension of cell therapies. Radiopharmaceutical cell labeling, firstly developed to track leukocytes, has been used successfully to evaluate the migration of cell therapies for myocardial diseases. A substantial rise in the amount of reports employing this methodology has taken place in the previous years. We will review the diverse radiopharmaceuticals, imaging modalities, and results of experimental and clinical studies published until now. Also, we report on current limitations and potential advances of radiopharmaceutical labeling for cell therapies in cardiac diseases.

## 1. Introduction

Cardiovascular ailments are still the greatest causes of morbidity and mortality in the world, with significant financial and social consequences [1, 2]. Despite recent medical and surgical advances in the past decades, currently there are no effective therapies to allow cardiac regeneration [3]. On this scenario, experimental studies have indicated that cell therapies may target cardiac regeneration in acute and chronic myocardial diseases [3]. Although clinical studies have already been carried out, the efficacy and potential mechanisms of cell therapies for cardiac diseases are still under continuous investigation [4–6]. Possible mechanisms of action of cell therapies include the secretion of paracrine factors that reduce cardiomyocyte death, improve local microcirculation, and decrease the amount of fibrous tissue, which may improve heart function [3].

Noninvasive imaging modalities have the potential of providing better understanding of the biological process and the effectiveness of cell therapies for cardiac diseases [7]. One of the main applications of these techniques is to track the migration of cell therapies [7]. Among the different imaging techniques available, Nuclear Medicine has become one of the most employed techniques, due to its favorable characteristics, such as the availability of different radiopharmaceuticals and its high sensitivity [8]. In this paper, we will review preclinical and clinical studies that used Nuclear Medicine to evaluate cell migration and discuss important issues in this area.

## 2. Use of Radiopharmaceuticals for Cell Labeling

In the past decades, labeled leukocyte scintigraphy has become an important method to locate sites of infection and inflammation in the body [9, 10]. The development of this method had been a key landmark in the history of Nuclear Medicine. Conventional techniques include two-dimensional planar scintigraphies and three-dimensional single photon emission computed tomography (SPECT). Additionally, SPECT images may be acquired together with a computed tomography, resulting in hybrid SPECT/CT images [11]. This technique allows a better location of the findings of Nuclear Medicine, thus increasing the sensitivity and specificity of the method [11].

A variety of labeling methods with radionuclides has been created and used to study cell distribution in the body [12]. Currently, technetium-99m (^99m^Tc) is the most commonly utilized radionuclide in the world, due to favorable properties such as its decay by gamma emission with an energy of 140 kev and a 6-hour half-life, optimum physical characteristics for SPECT, allowing images for up to 24 hours after injection [9]. Radionuclide indium-111 (^111^In) may also be used for cell labeling in SPECT, for example, through compounds ^111^In-oxine and ^111^In-tropolone [9].

The radionuclide fluorine-18 (^18^F) has a half-life of approximately 110 minutes and is the most frequently utilized in positron emission tomography (PET) and hybrid PET/CT, mainly in the radiopharmaceutical ^18^F-fluorodeoxyglucose (^18^F-FDG) [12]. PET has better spatial resolution than SPECT and allows the quantification of the standardized uptake value (SUV) [12, 13]. Zirconium-89 (^89^Zr) is another promising radionuclide for cell labeling in PET that has a 78.4-hour half-life and may allow cell tracking for two to three weeks [14].

Tracking cells with SPECT and PET may be separated in two strategies: direct and indirect [15]. Direct tracking is achieved by labeling cells with a radiotracer* in vitro* with subsequent cell administration [7, 15]. The most widely used radionuclides for direct labeling are ^99m^Tc and ^111^In to perform SPECT and ^18^F to perform PET [9, 16]. Indirect cell tracking may be achieved employing reporter gene/probe systems that have been the topic of exceptional reviews [8, 17]. For instance, a lentivirus may be used to deliver a reporter gene for expression of herpes simplex virus truncated thymidine kinase (TK) that catalyzes a reaction leading to the accumulation of the probe ^18^F-9-[4-fluoro-3-(hydroxymethyl)butyl]guanine derivatives (^18^F-FHBG) for PET imaging [17]. Another example of reporter gene is the Sodium Iodide Symporter (NIS), a cell surface protein expressed usually in thyroid cells, salivary glands, mammary glands, and choroid plexus, but not in organs such as the heart [18]. Cells overexpressing NIS will capture ^99m^Tc and iodine-123 (^123^I) for SPECT, as well as iodine-124 (^124^I) for PET, allowing the evaluation of viable cell homing in the heart after transplantation [18].

## 3. Preclinical Studies

### 3.1. Direct Cell Labeling

We identified 31 published articles that used direct cell labeling to track the migration and homing of cell therapies in preclinical models of heart diseases, all of them for myocardial infarction (Table 1).

#### 3.1.1. Effect on Cell Viability, Metabolic Activity, and Migration

Although the use of ^111^In radiopharmaceuticals allows cell tracking for longer periods in comparison to ^99m^Tc, it has high energy (171 and 245 keV), which leads to images of lower resolution and greater cell dose that may decrease cell viability [19–21]. ^111^In can affect the viability, metabolic activity, and migration of stem cells due to internalization of Auger electrons emitted at close distances. These electrons may lead to considerable toxicity to target cells reducing cell viability [10, 19–21].

Jin et al. carried out an interesting study where they evaluated the viability of bone marrow-derived mesenchymal stem cells (BM-MSCs) labeled with ^111^In [22]. Distinct samples with 5 × 10^6^ cells were labeled 0.1 to 18 MBq of ^111^In-tropolone. The authors reported that cells had 100% viability when incubated with up to 0.9 MBq, which corresponded to 0.14 Bq per cell.

Brenner et al. [19] reported the impact of labeling human CD34^+^ hematopoietic progenitor cells (HPCs) with ^111^In-oxine. HPCs (1 × 10^6^/mL) were incubated with 30 MBq of ^111^In-oxine for 1 hour to assess cell viability at 1, 24, 48, and 96 hours. Although no significant changes were observed at 24 hours after labeling, after 48 and 96 hours the number of dead cells increased. Furthermore, cell migration was quickly reduced after 24 hours.

Suhett et al. [23] studied the binding sites for ^99m^Tc in rat bone marrow mononuclear cells (BM-MNCs). BM-MNCs were labeled with 45 MBq of ^99m^TcO4-. After being labeled, cells were carefully disrupted and differentially centrifuged for organelle separation. Viability of the labeled cells was 93% and most of the radiation remained in the supernatant comprised of the cytosol and membrane bound ribosomes.

^18^F-FDG is regarded as the gold standard for the assessment of myocardial viability. ^18^F-FDG is a glucose analogue that enters cardiomyocytes through glucose transporters (GLUTs) such as GLUT1 and GLUT4. Within the cell, ^18^F-FDG suffers phosphorylation by hexokinase and converts to ^18^F-glucose 6-phosphate. Because it is not metabolized, it is retained within the cell. Preclinical studies made by Chan and Abraham reported that ^18^F-FDG caused no interference with proliferation of cardiac-derived stem/progenitor cells (CDCs) [7]. Similarly, Wolfs et al. found no significant changes to the ultrastructure and differentiation of mouse MSCs and rat multipotent adult progenitor cells [24].

Hexadecyl-4-[^18^F] fluorobenzoate (^18^F-HFB) is a lipophilic radiopharmaceutical that is absorbed through the cell membrane, allowing cell tracking by PET. Zhang et al. [25] compared the labeling of human peripheral blood-derived circulating progenitor cells (CPCs) with ^18^F-HFB and ^18^F-FDG in mice after myocardial infarction. Cells were injected close to the site of cardiac injury. The images were made in Micro-PET 10 min and 2 and 4 hours after injection. ^13^N-NH3 was used to outline the liver and the heart. Labeling with ^18^F-HFB showed no reduction in cell viability with 14.8–22.2 MBq of radioactivity in 2 × 10^6^; however, higher activities (185–259 MBq) resulted in significant cell death. After 24 hours, the reduction of viability in ^18^F-HFB-CPCs was 13.3%, whereas in controls it was 6.9%. After 5 days cell viability decreased for both groups: ^18^F-HFB-CPCs (10.4%) and ^18^F-FDG-CPCs (14.7%).

#### 3.1.2. Radionuclide Leakage and Labeling Efficiency

Quantifying* in vivo* cell transplant survival may be difficult, and radionuclide leakage is an important issue that should be taken into account [26]. Radionuclide leakage may occur from viable cells and cellular debris [26]. Many authors applied different* in vivo* experiments to determine cell death, radiolabel leakage, and cell survival [26, 27]. Another issue to be evaluated is the normal turnover of the cells, where one may label cells and administer them in order to study the clearance characteristics from viable cells that did not die* in vivo* [26].

Blackwood et al. [26] quantified the survival of BM-MSCs labeled with ^111^In transplanted into the canine myocardium. The authors also evaluated the clearance of lysed ^111^In labeled cells. Serial SPECT images were acquired after direct epicardial injection to determine the time-dependent radiolabel clearance. The average long biologic half-life for labeled cells was 74.3 hours and for lysed cells was 19.4 hours.

The labeling efficiency of direct labels differs between different methods and needs to be taken into account [28]. For instance, it has been reported that the labeling with ^99m^Tc-tropolone was more effective and stable in comparison to ^99m^Tc-hexamethylpropyleneamine oxime (^99m^Tc-HMPAO) [29]. In another example, Zhang et al. reported that ^18^F-HFB labeling showed a higher efficiency when compared with ^18^F-FDG [25].

#### 3.1.3. Biodistribution after Intravenous Injection

Kraitchman et al. [30] investigated the migration of BM-MSCs labeled with ^111^In-oxine, by intravenous route, 72 hours after the induction of lesion myocardial infarction in dogs. SPECT imaging was carried out up to 8 days after cell transplantation. Uptake on the same day of cell therapy was mainly restricted to the lungs in infarcted animals and control animals with low uptake in the heart. At 24 hours, uptake remained constant in the heart, decreased in the lungs, and increased in the liver and spleen.

Lutz et al. [31] studied the migration of systemically injected bone marrow-derived cells in mice after myocardial infarction. After induction of the infarction, animals received intramyocardial injections of stem cell factor (SCF) in peri-infarcted areas. Cells were labeled with ^111^In-oxine and injected in the tail vein 24 hours after the infarction. Animals were sacrificed and hearts removed for analysis in a gamma counter 24 or 72 hours later. The analysis indicated that intramyocardial injections of SCF significantly increased myocardial uptake in comparison with infarcted animals that received saline injections and with sham-operated animals at both time points.

Garikipati et al. [32] investigated the efficacy of therapy with fetal cardiac mesenchymal stem cells (FC-MSCs) in rats after myocardial infarction. FC-MSCs were isolated and cultured from fetal rat hearts. Seven days after the induction of the lesion, mice were divided into FC-MSC or saline group. Cells were labeled with ^99m^Tc-HMPAO and injected into the tail vein. Multipinhole gated SPECT/CT was carried out six hours after the intravenous infusion and ^99m^Tc labeled cells were mainly present in the lungs, with focal homing in the heart.

#### 3.1.4. Biodistribution after Intraventricular Injection

Brenner et al. [19] performed intraventricular injections of human HPCs into the left ventricular cavity of rats after myocardial infarction. SPECT was performed 1, 24, 48, and 96 hours after transplantation. Liver, kidneys, and spleen combined had 37% and lungs 17% of whole body uptake 1 h after cell transplantation. Twenty-four hours after the injection, lung uptake was no longer detected, while homing to the liver, kidneys, and spleen increased to 57%. Only 1% of the injected activity was found in the heart of transplanted animals.

Aicher et al. investigated the transplantation of ^111^In-oxine labeled endothelial progenitor cells (EPCs) into rats after myocardial infarction [33]. Labeled cells were delivered in the tail vein or in the left ventricular cavity. Pinhole SPECT was performed after cell administration. Total uptake in the liver, kidneys, and spleen was 71% after 96 hours, while myocardial uptake was only 1-2% after intravenous injection and 3–5% after intraventricular cavity infusion.

Barbash et al. evaluated the effectiveness and feasibility of systemic administration of BM-MSCs in rats following myocardial infarction. Cells were labeled by incubation with ^99m^Tc-HMPAO [34]. Three injection methods were studied. The first approach was by infusion of BM-MSCs in the femoral vein. In the second strategy, BM-MSCs were infused directly into the left ventricle. In the third group, cells were injected into the right ventricle, but all animals died from pulmonary embolism. Images were acquired 4 hours after the infusion and indicated that rats with myocardial infarction had higher uptake of ^99m^Tc labeled cells in the heart than sham animals. Moreover, intravenous infusion resulted in lower myocardial homing due to pulmonary cell retention.

#### 3.1.5. Biodistribution after Intramyocardial Injection

Zhou et al. [35] investigated the distribution of rat embryonic cardiomyoblasts (H9c2) cells after labeling with ^111^In-oxine rats after myocardial infarct. Cells were intramyocardially transplanted around the infarcted region immediately after induction of the lesion and SPECT images acquired 2, 24, 48, 72, and 96 hours. The authors reported that cell uptake was detected in the injection site up to 96 hours after administration.

Shen et al. [36] used magnetic resonance imaging (MRI) and SPECT imaging to monitor H9c2 cell transplantation in rats after myocardial infarction. Myocardial infarction was induced and ^111^In labeled cells were injected in regions close to the injured site. MRI was performed 5–7 days after SPECT images. Through a coregistration algorithm, it was possible to carry out the fusion of SPECT-MRI images. The authors were able to monitor the uptake of ^111^In-oxine labeled cells and the perfusion in ^99m^Tc-sestamibi images.

Tran et al. [37–39] evaluated in a series of studies the migration of ^111^In-oxine labeled BM-MSCs in rats one to four months after myocardial infarction in rats. Cells were injected in the infarcted areas. Cell distribution was compared with ^99m^Tc-sestamibi imaging of myocardial perfusion using a 17-segment division of the left ventricle. The authors concluded that BM-MSCs homing was heterogeneous and did not match in all occasions the infarcted regions [37–39].

Wisenberg et al. [40] evaluated dogs using both imaging of ^111^Indium-tropolone labeled cells and late gadolinium enhancement cardiac MRI for up to 12 weeks after a 3-hour coronary occlusion. The animals were injected with BM-MSCs and imaged at day 0 (surgery) and after 4, 7, 10, and 14 days. SPECT imaging indicated an effective biological clearance half-life from the injection site of ~5 days, while cardiac MRI demonstrated a pattern of progressive infarct reduction over 12 weeks.

Terrovitis et al. [41] labeled rat CDCs with ^18^F-FDG to monitor cell therapy in rats after myocardial infarction. CDCs were injected intramyocardially. In other groups of animals, the effects of fibrin glue, bradycardia (by adenosine injection), and induction of cardiac arrest on cell homing were investigated. One hour after cell transplantation without additional measures, PET indicated that mean myocardial homing was 17.8%. Adenosine injection was able to decrease the heart rate and double cell mean cell homing to 35.4%. A comparable enhancement in cell homing was seen when the authors applied fibrin glue epicardially and mean cell homing increased to 37.5%. However, the greatest increase was seen after induction of cardiac arrest, when mean homing increased to 75.6%.

Lang et al. [42, 43] studied the distribution of ^18^F-FDG labeled murine embryonic stem cells (ESCs) or fibroblasts in C57BL6/N mice after myocardial infarction, five minutes after the infarct ESCs or fibroblasts were injected intramyocardially [42, 43]. Images were made in a preclinical PET. The authors reported that the percentages of uptake in the heart were 5.2–5.3% after 25 minutes, 4.8–5.0% after 1 hour, and 5.6–5.7% after 2 hours.

Danoviz et al. assessed the transplantation of adipose tissue-derived stem cells (ADSCs) with two biopolymers, fibrin and collagen, in murine model of acute myocardial infarction [27]. Cells were labeled with ^99m^Tc-HMPAO. Twenty-four hours after induction of the lesion, the animals were injected with cells suspended in 100 mL of carrier by intracoronary route. Cells were infused in the border of the lesion with fibrin, collagen, or culture medium. Radioactivity counting of the organs revealed high levels of radioactivity in the liver, kidneys, and lungs. Both biopolymers increased cellular retention, but the collagen group showed higher uptake (26.8%) when compared to fibrin and culture medium (13.7% and 4.84%, resp.).

Mitchell et al. [44, 45] and Sabondjian et al. [46] assessed the migration of EPCs in canine models of myocardial infarction up to 7 days after induction of the lesion. EPCs were labeled with ^111^In-tropolone and injected by epicardial and endocardial routes. SPECT imaging was performed up to 15 days after cell transplantation. The authors reported that cell homing occurred in hypoperfused areas and that epicardial and endocardial injections led to similar uptake.

Maureira et al. [47] developed an* in vivo* technique with pinhole SPECT to monitor stem cell migration after myocardial infarction in rats. After coronary occlusion, autologous BM-MSCs were labeled with ^111^In-oxine. An intramyocardial injection was administered in the infarcted region. Two days after the procedure, ^99m^Tc-sestamibi was injected to compare homing of ^111^In labeled cells and myocardial perfusion. Left ventricle perfusion and function in all animals were monitored 2 days before cell therapy and 1–6 months after therapy using a pinhole gated SPECT. Significant improvements in cardiac perfusion were observed in injured areas and also in areas not transplanted.

Kim et al. [48] studied the homing of ADSCs after direct labeling with ^124^I-hexadecyl-4-tributylstannylbenzoate (^124^I-HIB) or ^18^F-FDG in rats after myocardial infarction. Cells were labeled with ^124^I-HIB or ^18^F-FDG. An intramyocardial injection was performed in the infarct site. ^124^I-HIB labeled cells were seen at the infarct area and monitored for up to 3 days in lesioned animals. The authors reported that labeling efficiency with ^124^I-HIB was higher than with ^18^F-FDG, indicating it could be a good method to monitor stem cell homing.

Elhami et al. [49] investigated the migration of ^18^F-FDG labeled ADSCs after myocardial infarction in rats. Labeling was carried out with ^18^F-FDG. Immediately after the infarct induction, cell transplantation was carried out by intramyocardial, intraventricular, or intravenous route. In another group, cells were injected intramyocardially 7 days after the infarct. The authors reported that the intravenous route led to lower cell homing in the heart (1.2% of infused ADSCs) 4 hours after cell transplantation. Intraventricular injection led to an uptake of 3.5% in the heart, while intramyocardial injection led to the highest myocardial cell homing (14%). Interestingly, in the group that received an intramyocardial cell injection 7 days after the myocardial infarction, cell homing was lower (4.5%) than the group that received cells immediately after the infarct induction.

#### 3.1.6. Biodistribution after Intracoronary Injection

Qian et al. [50] determined the distribution of BM-MNCs after myocardial infarction in Chinese mini-pigs. Cells were labeled with ^18^F-FDG and injected intramyocardially 7 days after the infarct. One hour after cell transplantation, 6.8% of the whole body uptake was located in the infarct site. Liver and spleen showed more than 90% of the uptake.

Doyle et al. [51] tracked CPCs in pigs after acute myocardial infarction. CPCs were labeled with ^18^F-FDG. One group received CPCs divided into 3 cycles after a balloon catheter was positioned and inflated in the lesioned artery. A second group received a single bolus infusion of CPCs without balloon inflation. The authors reported that one hour after cell transplantation the group that received the infusion in 3 cycles with balloon occlusion had lower uptake in the heart than the group that received a single bolus injection (8.7% versus 17.8%, resp.). The majority of activity (>60%) was concentrated in the lungs after 1 hour in both groups, and there was moderate uptake in the liver and spleen.

Keith et al. [52] investigated the impact of using intracoronary human CDC injection on cell homing in a pig model of myocardial infarction. Cells were injected with or without balloon inflation after labeling with ^111^In-oxine. SPECT was carried out 24 hours after cell transplantation. The authors reported that the injection with balloon occlusion led to similar myocardial homing as the one without balloon occlusion (5.41% versus 4.87%, resp.) and concluded that the risk involved in the coronary occlusion approach would not be warranted.

Hou et al. evaluated the distribution of peripheral blood mononuclear cells (PB-MNCs), labeled with ^111^In, in pigs after myocardial infarction. The lungs had 1%, 3%, and 3% of the uptake, while myocardial uptake was 2.6%, 3.2%, and 11% after intracoronary, interstitial retrograde coronary venous, or intramyocardial injections, respectively.

Tossios et al. [53] monitored the distribution of BM-MNCs following induction of myocardial infarction in pigs. After labeling with ^111^In-tropolone, cells were injected by intramyocardial or by intracoronary route with or without balloon occlusion. One hour after injection, 20.7%, 4.1%, and 6.1% of the uptake were located in the heart after intramyocardial, intracoronary without balloon, and intracoronary with balloon infusions, respectively. Twenty-four hours later, myocardial uptake was 15.0%, 3.0%, and 3.3%, respectively. The lungs, liver, and spleen had 50%, 10%, and 5% of the uptake in the whole body, respectively.

Mäkelä et al. [54] evaluated the migration of BM-MNCs in a pig model of myocardial infarction. Cells were labeled with ^111^In-oxine and transplanted by intramyocardial or intracoronary routes 30 minutes after induction of the lesion. SPECT was acquired 2 and 24 hours after cell transplantation and biopsies from different organs were also performed to allow gamma counting. The authors reported that the intracoronary injection led to <15% of the cardiac uptake observed after intramyocardial injection, while lung uptake after intramyocardial injection was <15% of the pulmonary uptake observed after intracoronary infusion.

Forest et al. studied a preclinical model of myocardial infarction in pigs [55]. Seven days after induction of the lesion, BM-MNCs were labeled with ^99m^Tc. Animals were divided into three groups: control group, intracoronary injection, and intravenous injection of ^99m^Tc labeled cells. Intravenous administration led to higher cell accumulation in the lungs, while intracoronary injection led to greater myocardial uptake.

### 3.2. Indirect Radiolabeling: Reporter Gene/Probe Systems

Reporter gene/probe imaging for SPECT and PET has been applied to evaluate the survival of transplanted cells in animal models of cardiac diseases [8]. Some of the disadvantages of using reporter genes include the possible immunogenicity of the viral reporter gene, which limits the application of the technique in humans [7]. Moreover, the stability of transfection and expression must be improved and the potential interference with stem cell function and differentiation from vector transfection or transduction must be minimized [56]. We identified 9 published articles that used indirect cell tracking to evaluate the migration and homing of cell therapies in preclinical models of heart diseases, all of them for myocardial infarction (Table 2).

#### 3.2.1. Biodistribution after Intramyocardial Injection

Gyöngyösi et al. [57] used reporter gene imaging to monitor the migration of BM-MSCs in a pig model of myocardial infarction. Cells transfection was performed with a lentivirus for expression of TK. Sixteen days after the infarction, a group of animals received BM-MSCs by intramyocardial injection. Then, ^18^F-FHBG was injected 30 hours and 7 days after cell transplantation for* in vivo* imaging. The authors reported that there was a decrease in myocardial uptake of ^18^F-FHBG after 7 days in comparison with the 30-hour images, as well as mild increase in pericardial and pleural uptake.

Terrovitis et al. [58] transfected rat CDCs with a lentivirus to express the NIS gene.* In vivo* images were obtained after intramyocardial cell injection in mice after myocardial infarction. An injection of ^99m^Tc for SPECT imaging or ^124^I for PET imaging was used to evaluate the expression of NIS gene in transplanted CDCs. The authors were able to detect the transplanted CDCs with a threshold of approximately 10^5^ cells. Cell homing was seen up to 6 days after CDC transplantation but less than 5% of cells remained in the heart, due to migration to the lungs and systemic circulation.

Lee et al. [59] investigated the homing of canine iPSCs after cell transplantation in dogs. Cells were injected intramyocardially 30 minutes after the induction of myocardial infarction. The authors injected an activity of approximately 536 MBq of ^18^F-FHBG and carried out PET/CT 8 hours after cell transplantation. Imaging revealed cell homing to the anterior myocardial wall.

Liu et al. [60] and Lan et al. [61] analyzed the migration of human CDCs in rats after myocardial infarction in severe combined immunodeficiency (SCID) Beige mice. A total of 1 × 10^6^ cells transfected with a TK reporter gene were injected by intramyocardial route immediately after the induction of myocardial infarction. On days 1, 7, 14, 21, and 28 after cell therapy, an activity of 7.4 MBq of ^18^F-FHBG was injected to allow PET imaging. A gradual decrease in the amount of surviving cells was noticed during the follow-up. Interestingly, the authors reported that early cell homing predicted ensuing functional improvement [60].

Using reporter genes, Templin et al. [62] were able to monitor human induced pluripotent stem cells (iPSCs) in pigs after myocardial infarction. Cells were labeled 90 minutes before injection with 100 MBq of ^123^I and a volume of 250 *μ*L was injected in three regions of the animals' hearts. The anterior wall of the left ventricle received 50 million cells of human MSCs. The lateral and septal walls received 50 million NIS-positive [NIS(pos)] human iPSCs or 50 million NIS(pos) human iPSCs mixed with 50 million human MSCs. ^99m^Tc-tetrofosmin was intravenously injected to assess myocardial perfusion. Images were acquired for 5 minutes in SPECT/CT equipment. Images were acquired up to 15 weeks after cell transplantation through an intracoronary injection of ^123^I. No uptake was seen outside the heart and NIS(pos) human iPSCs were detected in the site of injections, indicating successful cell homing.

Yan et al. [63] assessed the distribution of BM-MSCs in nude mice 10 minutes after induction of myocardial infarction. Cells transfected with a TK gene were injected intramyocardially after induction of the lesion. On the same day and 3 and 7 days after cell transplantation, ^18^F-FHBG was injected and PET was carried out. The authors described that the highest myocardial uptake occurred 3 days after cell therapy and that infarcted animals had higher homing than control animals.

Pei et al. [64] evaluated the homing of BM-MSCs in rats after myocardial infarction. Immediately after the lesion, cells were intramyocardially injected. Two, 3, and 7 days after cell transplantation, ^18^F-FHBG was injected to allow cell tracking. The authors reported that myocardial uptake could be seen up to 7 days following cell therapy, and homing was mostly distributed to the liver, lungs, intestines, stomach, and spleen.

Lee et al. [65] studied the distribution of ADSCs transfected with the NIS gene in dogs following myocardial infarction. NIS expressing ADSCs were intramyocardially injected 7 days after the infarct induction. ^99m^TcO4- was injected at 2 hours and 1, 2, 5, 7, 9, and 12 days after cell transplantation. The authors reported that cell homing was identified in the apex and lateral wall of the left ventricle, reached its peak at 2 days, and was seen until 9 days after cell transplantation.

## 4. Clinical Trials

### 4.1. Direct Cell Labeling

We have found 18 published articles in English regarding 17 different trials that employed radionuclides to track cell therapies for cardiac diseases, with a total of 293 treated patients (Table 3). All studies used direct labeling methods.

#### 4.1.1. Biodistribution after Intracoronary Injection

Caveliers et al. [66] conducted a cell therapy trial with eight chronic ischemic heart disease patients. They reported that infusion of CD133^+^ selected PB-MNCs labeled with ^111^In-oxine is a safe and feasible procedure. They also performed ^99m^Tc-MIBI SPECT for evaluation of myocardial perfusion and compared it to cell migration. Uptake in the heart was 6.9% to 8% and 2.3% to 3.2% after 2 and 12 hours, respectively.

Kurpisz et al. [67] studied the migration of BM-MNCs in 3 patients with acute myocardial infarction. Cells were labeled with ^111^In-oxine and injected by intracoronary route. Nuclear Medicine imaging was carried out 24 hours after cell transplantation. The authors reported that 2.6–11.0% of the uptake was seen in the heart, 12.3–56.7% in the liver, and 5.2–12.6% in the spleen.

Schots et al. [68] evaluated 13 patients with nonacute myocardial infarction who received CD133^+^ cells labeled with ^111^In-oxine by intracoronary transplantation. Subjects had uptake of 6.9 to 8.0% in the myocardium in 2-hour images and 2.3 to 3.2% in 12-hour images.

Schächinger et al. [69] included 20 patients with ischemic myocardial disease that had myocardial viability confirmed by PET and intracoronary Doppler. The time of coronary injury to BM-MNC therapy ranged from 5 days to 17 years. After administration of ^18^F-FDG labeled cells, the average myocardial uptake in the first 24 hours was higher in subjects with acute myocardial infarction and gradually decreased in subjects treated in an intermediate or chronic phase. The authors concluded that the low viability of the lesioned myocardium and the reduction of coronary flow reserve were important predictors in the proangiogenic potential of progenitor cells.

Dedobbeleer et al. [70] published a study of 12 patients with nonacute myocardial infarction. Five patients were in the control group and 7 patients had CD34^+^ cells labeled with ^18^F-FDG. After an hour of injection, 3.2% of the radioactivity was observed in the myocardial infarction zone.

Blocklet et al. [71] evaluated the injection of PB-MNCs labeled with ^111^In-oxine and ^18^F-FDG in 6 patients with acute myocardial infarction. The double labeling allowed monitoring of cell with high sensitivity and resolution with PET and performing late images with ^111^In. Mean uptake in the myocardium after 1-hour infusion of PB-MNCs was 5.5% by PET, while in images with ^111^In-oxine at 19 hours and 43 hours only 1 patient had myocardial uptake.

#### 4.1.2. Comparison of Biodistribution of Intracoronary and Intravenous Injection

Hofmann et al. [72] carried out a cell therapy trial 5 to 10 days after a myocardial infarction in 9 patients using CD34^+^ BM-MNCs. Of the total amount of injected cells, 5% were labeled with ^18^F-FDG. The patients were divided into 3 protocols. In the first protocol, 3 patients received unselected BM-MNCs by intracoronary route and underwent PET imaging 55 to 75 minutes after infusion. In a second protocol, 3 patients initially received 5% of the unselected BM-MNCs by intravenous route, followed by a first PET 50 to 60 minutes after cell transplantation, and then received the remaining 95% of unselected BM-MNCs by intracoronary route, followed by a second PET 60 to 70 minutes later. In a third protocol, 3 patients received immunomagnetically enriched CD34^+^ cells by intracoronary route and underwent PET imaging 60 to 75 minutes after cell injection. In the first protocol, homing varied from 1.3% to 2.6%. In the second group, there was no detectable myocardial homing after the initial intravenous infusion, but homing increased to 1.8 to 5.3% after intracoronary injection. In the third group, in which CD34^+^ cells were injected by intracoronary route, cell homing was higher, varying from 14% to 39%.

Kang et al. [73] published a report in which 20 patients with recent or old myocardial infarctions received PB-MNCs labeled with ^18^F-FDG. The PB-MNCs were collected by apheresis after mobilization with granulocyte colony stimulating factor (G-CSF). Seventeen of the patients received cells by intracoronary route and 3 patients by intravenous route. The mean efficiency of cell labeling with ^18^F-FDG was of 72% and a total activity of 44.4 to 175 MBq was injected through a catheter after stent implantation in the infarcted artery. PET/CT images were obtained 2, 4, and 24 hours after injection. Two hours after intracoronary injection, 1.5% of the infused cells were present at the lesioned area. Delayed images up to 20 hours indicated prolonged accumulation of the cells in heart tissue. Intravenous infusion of the labeled PB-MNCs revealed high pulmonary trapping and showed no significant activity in the heart.

Goussetis et al. [74] studied 8 subjects with chronic ischemic heart disease undergoing CD133^+^ and CD133^−^CD34^+^ selected BM-MNC transplantation by intracoronary infusion. Cells were labeled with ^99m^Tc and scintigraphies acquired 1 and 24 hours after injection indicated cardiac uptake of 9.2% and 6.8%, respectively. Reevaluation with coronary angiography and echocardiography in 6 patients after 3 months of cell therapy revealed no complications.

Penicka et al. [75] included 10 patients, 5 of them with acute myocardial infarction and the other 5 with nonacute myocardial infarction. All patients received BM-MNCs labeled with ^99m^Tc-HMPAO and myocardial uptake was analyzed 2 and 20 hours after injection. There was a lack of uptake 20 hours after transplantation in subjects with acute myocardial infarction.

A randomized study of 30 subjects with acute myocardial infarction, published by Silva et al. [76] and Moreira et al. [77], compared the distribution and retention pattern of ^99m^Tc-HMPAO labeled BM-MNCs after anterograde intra-arterial or retrograde intravenous coronary routes. The early and late retention of labeled cells, evaluated in 4 and 24 hours SPECT images after injection, were higher in the group that received cells by coronary anterograde, regardless of the presence of microcirculation obstruction. Early and late retention were, respectively, 7.06% and 6.38% in the intra-arterial group and 1.4% and 0.99% in the intravenous group.

Musialek et al. [78] compared the cell transplant management techniques: perfusion technique catheter (PC) and the over-the-wire coronary occlusion technique (OTW). Thirty-four patients who suffered myocardial infarction were randomly assigned to PC or OTW infusion of autologous bone marrow CD34^+^ cells labeled with ^99m^Tc-HMPAO. One hour after infusion, the images obtained by SPECT indicated the activity of 4.86% and 5.05% in the myocardium after OTW and PC injections, respectively. The authors concluded that although the efficacy of cell delivery did not differ between infusion methods, PC infusion offered a more physiological alternative and avoided causing OTW ischemic episodes. The same group performed another study evaluating the migration of intracoronary injected ^99m^Tc-HMPAO labeled bone marrow CD34^+^ cells in subjects after myocardial infarction. The authors described that, one hour after cell transplantation, mean cardiac uptake was 5.2% [79].

Our group published a study with 6 Chagasic cardiomyopathy patients who received intracoronary injection of ^99m^Tc labeled BM-MNCs [80]. SPECT images performed 1, 3, and 24 hours after administration of the labeled cells revealed a myocardial uptake of 5.4%, 4.3%, and 2.3%, respectively. Such decrease in relative myocardial uptake could be related to leakage of ^99m^Tc from labeled cells and not to a reduction in the number of cells. We also observed that the cell distribution was heterogeneous and limited and was related with the pattern of myocardial perfusion.

Kollaros et al. [81] compared images obtained from the perfusion study with ^201^Tl and images after intracoronary infusion of BM-MNCs labeled with ^99m^Tc-HMPAO. In the thirteen patients, images were complementary and revealed accurate localization of cells in the lesioned area. There was intense cell accumulation in areas without viability as evaluated by ^201^Tl scintigraphy. The percentage (83.2%, ranging from 56.4 to 97.2%) of the infarcted area that had retained cells was determined by merging ^99m^Tc and ^201^Tl images.

#### 4.1.3. Comparison of Biodistribution after Intracoronary and Transendocardial Injection

Vrtovec et al. [82] included a total of 40 patients, where 20 received ^99m^Tc labeled BM-MNCs by intracoronary and another 20 by transendocardial route. The relative uptake after 18 hours after injection was 4.4% and 19.2% in intracoronary and transendocardial routes, respectively.

Haddad et al. [83] included thirty-seven patients with nonischemic dilated cardiomyopathy. On average, 75 × 10^6^ CD34^+^ PB-MNCs were labeled with ^99m^Tc-HMPAO and infused via transendocardial route. SPECT images were acquired 2 and 18 hours after infusion to assess the homing and cellular distribution as well as detect cell migration potential. Twenty-eight patients consented to further myocardial homing imaging. In those patients, the stem cells homing rate had a median value of 11.4% (range 3.8%–22.3%).

## 5. Alternative Approaches to Cell Tracking

Besides radionuclide labeling, different techniques may be used to study cell distribution* in vivo*. Fluorescence imaging (FLI) and bioluminescence imaging (BLI) have been effectively employed to track cells in preclinical studies of cell transplantation for cardiac diseases [84, 85]. Nevertheless, factors such as the limited tissue penetration of light hinder the clinical application of FLI and BLI [86]. Superparamagnetic iron oxide nanoparticles (SPIONs), originally created to detect liver tumors in patients after intravenous infusion, were adapted for preclinical exogenous cell labeling, which allowed the study of cell migration for weeks following transplantation with exceptional resolution and morphologic correspondence with MRI [87]. Early clinical studies have been conducted in studies of cell therapies for noncardiac diseases [88–92]. Nonetheless, SPION labeling has restrictions of other exogenous contrasts, for instance, the possibility of dilution with cellular division and of stem cell phagocytosis by macrophages. Moreover, there are differing data on the burden of nanoparticle cell labeling in biological properties [93–96], and exogenous SPION cell labeling has only been approved for research applications.

Due to these factors, radiopharmaceutical labeling continues to be a relevant technique for the assessment of stem cell distribution* in vivo* [7]. It allows more accurate definition of cell location and the combination of Nuclear Medicine with CT or MRI enables the study of diverse characteristics, for example, (1) comparison of cell migration with structural and functional results and (2) the outcome of different cell doses and injection methods on cell homing.

## 6. Impact of the Route of Administration

Radiopharmaceutical cell tracking has already increased understanding of cell migration in preclinical and clinical studies of cell therapies for cardiac diseases. Among other conclusions, preclinical [55] and clinical [54, 73] studies indicated that intravenous infusions of BM-MNCs and PB-MNCs lead to lower cardiac homing in comparison with intracoronary injections. On the other hand, intramyocardial injection of PB-MNCs [97] and BM-MNCs [53, 54] led to greater cardiac homing of transplanted cells in comparison to intracoronary infusion in preclinical studies. Similarly, transendocardial injection of BM-MNCs led to greater homing in comparison to intracoronary infusion in subjects with nonischemic dilated cardiomyopathy [82].

Even though there have been preclinical and clinical studies investigating the potential of MSC transplantation for cardiac diseases, to our knowledge, no clinical studies yet have tracked MSC migration with noninvasive imaging. Moreover, clinical trials of radiopharmaceutical cell tracking remain restricted to PB-MNC and BM-MNC trials.

Nevertheless, it is still unclear if more intense myocardial homing is important to improve the outcome of cell therapies for cardiac diseases. Different groups have suggested that, instead of differentiation into cardiac cells, the mechanisms of stem cell therapies may be at least partially due to interactions between injected and host cells, such as the secretion of trophic factors [98]. For example, BM-MSCs may assume distinctive phenotypes after receiving stimuli from proinflammatory cytokines or when submitted to a hypoxic milieu* in vitro* [98].

As previously mentioned, intravenously injected stem cells may suffer pulmonary entrapment [99]. The lungs may characterize an obstacle for cell migration [99] but might also be essential for the triggering of stem cell responses, before their homing to the heart. Lee et al. [100] reported that an increased production of the tumor necrosis factor inducible gene 6 protein (TGS-6) in BM-MSCs is entrapped in the lungs after intravenous injection in mice following acute myocardial infarction. Their report suggested that BM-MSCs were stimulated in the lungs to produce TGS-6, which controlled myocardial inflammatory response.

## 7. Conclusion

Methods for cell tracking with radioisotopes are feasible and efficient and different studies have used it to monitor migration in cell therapies for cardiac diseases. These techniques provide validated quantifications of cell retention in different organs and the dynamics of cell distribution in the whole body. However, additional reports are needed to increase the knowledge of the mechanisms responsible for cell migration and homing and their relationship with possible structural and functional outcomes of cell transplantation for cardiac diseases.

## Figures and Tables

**Table 1:** Preclinical studies that used direct radiopharmaceutical labeling for cell therapies in models of myocardial infarction.

Study reference	Radiopharmaceutical used for cell tracking	Time window of cell injection after lesion induction	Animals	Cell type	Route(s)	Number of cells injected	Radionuclide activity	Time points of analysis after cell therapy
Aicher et al., 2003 [33]	^111^In-oxine	24 h	Athymic nude rats	Human EPCs	Intravenous Intraventricular	1 × 10^6^	15 Bq/cell	1, 24, 48, and 96 hours
Barbash et al., 2003 [34]	^99m^Tc-HMPAO	2 days or 10–14 days	Athymic nude rats	Rat BM-MSC	Intravenous Intraventricular	4 × 10^6^	Not specified	Not specified
Brenner et al., 2004 [19]	^111^In-oxine	24 hours	Athymic nude rats	Human HPCs	Intraventricular	Not specified	30 Bq/cell	1, 24, 48, and 96 hours
Zhou et al., 2005 [35]	^111^In-oxine	Immediately	Sprague-Dawley rats	Rats H9c2	Intramyocardial	3-4 × 10^6^	3,7 Bq/cell	2, 24, 48, 72, and 96 hours
Kraitchman et al., 2005 [30]	^111^In-oxine	72 hours	Dogs	Canine BM-MSCs	Intravenous	1.6 × 10^8^	0,4 Bq/cell	Same day to 8 days
Hou et al., 2005 [97]	^111^In-oxine	6 days	Pigs	Human PB-MNCs	Intracoronary Intramyocardial IRV	1 × 10^7^	1,85 Bq/cell	1 hour
Tran et al., 2006 [37]	^111^In-oxine	3 months	Wistar rats	Rat BM-MSCs	Intramyocardial	2 × 10^6^	7,5 Bq/cell	2 days
Tran et al. 2006 [38]	^111^In-oxine	1 month	Wistar rats	Rat BM-MSCs	Intramyocardial	2 × 10^6^	7,5 Bq/cell	7 days
Shen et al., 2007 [36]	^111^In-oxine	Immediately	Sprague-Dawley rats	Rat embryonic cardiomyoblasts	Intramyocardial	3-4 × 10^6^	3,7 Bq/cell	30 minutes
Tran et al., 2007 [39]	^111^In-oxine	4 months	Wistar rats	Rat BM-MSCs	Intramyocardial	2 × 10^6^	7,5 Bq/cell	48 h
Qian et al., 2007 [50]	^18^F-FDG	7 days	Chinese mini-pigs	Porcine BM-MNCs	Intracoronary	1.0 × 10^9^	0,185 Bq/cell	1 hour
Doyle et al., 2007 [51]	^18^F-FDG	48 hours	Pigs	Porcine CPCs	Intracoronary	3 × 10^7^	Not specified	1 hour
Lutz et al., 2008 [31]	^111^In-oxine	24 h	C57BL/6 mice	Murine bone marrow-derived cells	Intravenous	1 × 10^6^	0,037 Bq /cell	24 and 72 hours
Tossios et al., 2008 [53]	^111^In-tropolone	5 days	Pigs	Porcine BM-MNCs	Intracoronary Intramyocardial	1 × 10^8^	Not specified	Immediately; 1–24 hours
Blackwood et al. 2009 [26]	^111^In-tropolone	Immediately	Dogs	Canine BM-MSCs	Intramyocardial	3.08 × 10^7^	1,7 Bq/cell	Same day and 2 weeks
Terrovitis et al., 2009 [41]	^18^F-FDG	Immediately	Wistar Kyoto rats	Rat CDCs	Intramyocardial	2 × 10^6^	Not specified	Not specified
Mäkelä et al., 2009 [54]	^111^In-oxine	30 minutes	Pigs	Porcine BM-MNCs	Intramyocardial Intracoronary	2 × 10^8^	0,185 Bq/cell	2 and 24 hours and 6 days
Mitchell et al., 2010 [44]	^111^In-tropolone	Same day and 7 days	Dogs	Canine EPC	Epicardial Endocardial	2.8 × 10^7^	0,1 Bq/cell	30–40 minutes 4 and 10 and up to 15 days
Forest et al., 2010 [55]	^99m^Tc	5 days	Pigs	BM-MNCs	Intravenous Intracoronary	1 × 10^7^ (intracoronary) 1 × 10^8^ (intravenous)	Not specified	1 and 24 hours
Danoviz et al., 2010 [27]	^99m^Tc-HMPAO	24 hours	Lewis rats	Rat ADSCs	Intramyocardial	1 × 10^6^	Not specified	Not specified
Sabondjian et al., 2012 [46]	^111^In-tropolone	Not specified	Dogs	Canine EPCs	Intramyocardial	Not specified	Not specified	Immediately; 4 and 10 days
Zhang et al., 2012 [25]	^18^F-HFB ^18^F-FDG	14 days	Sprague-Dawley rats	Human CPCs	Epicardial Endocardial	2 × 10^6^	7,4 to 11,1 Bq/cell	10 min, 2 and 4 hours
Mitchell et al., 2013 [45]	^111^In-tropolone	First: 4 hours or 7 days/second: 4 weeks later	Dogs	Canine EPCs	Intramyocardial	3 × 10^7^	0,1 Bq/cell	Same day and 4, 10, and 15 days
Maureira et al., 2013 [47]	^111^In-oxine	4 months	Wistar rats	Rat BM-MSCs	Intramyocardial	2 × 10^6^	7,5 Bq/cell	48 hours
Lang et al., 2013 [42]	^18^F-FDG	5 minutes	C57BL6/N wild-type mice	Murine ESCs	Intramyocardial	3 × 10^6^	0.8 Bq/cell	25 minutes and 2 hours
Elhami et al., 2013 [49]	^18^F-FDG	Immediately or 7 days	Lewis rats	Rat ADSCs	Intramyocardial Intraventricular, intravenous	4.5–6.0 × 10^6^	20 Bq/cell	4 hours
Garikipati et al., 2014 [32]	^99m^Tc-HMPAO	7 days	Sprague-Dawley rats	Rat FC-MSC	Intravenous	2 × 10^6^	37 Bq/cell	6 hours
Lang et al., 2014 [43]	^18^F-FDG	5 minutes	C57BL6/N wild-type mice	Murine ESCs and fibroblasts	Intramyocardial	3 × 10^6^	0,8 Bq/cell	2 hours
Kim et al., 2015 [48]	^124^I-HIB ^18^F-FDG	Not specified	Sprague-Dawley rats	Rat ADSCs	Intramyocardial	5 × 10^6^	0,2 to 0,3 Bq/cell	1 day (^18^F-FDG) and 9 days (^124^I-HFB)
Keith et al., 2015 [52]	^111^In-oxine	1-2 months	Pigs	Human CDCs	Intracoronary	1 × 10^7^	27.7 Bq/cell	24 hours
Bansal et al., 2015 [14]	^89^Zr	1 hour	Mice	Human MSCs	Intramyocardial	2 × 10^5^	0.37 Bq/cell	2, 5, and 7 days

^111^In: indium-111; ^18^F: fluorine-18; ^89^Zr: zirconium-89; ^99m^Tc: technetium-99m; ADSCs: adipose tissue-derived stem cells; BM-MNCs: bone marrow mononuclear cells; BM-MSCs: bone marrow mesenchymal stem cells; CDCs: cardiac-derived stem/progenitor cells; CPCs: circulating progenitor cells; EPCs: endothelial progenitor cells; ESCs: embryonic stem cells; FC-MSCs: mesenchymal stem cells derived from rat fetal heart; FDG: fluorodeoxyglucose; HFB: hexadecyl-4-fluorobenzoate; HIB: hexadecyl-4-tributylstannylbenzoate; HMPAO: hexamethylpropyleneamine oxime; IRV: interstitial retrograde coronary venous; PB-MNCs: peripheral blood mononuclear cells.

**Table 2:** Preclinical studies that used indirect radiopharmaceutical tracking for cell therapies in models of myocardial infarction.

Study reference	Radiopharmaceutical used for cell tracking	Time window of cell injection after lesion induction	Animals	Cell type	Route(s)	Number of cells injected	Time points of analysis after cell therapy
Gyöngyösi et al., 2008 [57]	^18^F-FHBG	16 days	Pigs	Porcine BM-MSCs	Intravenous	1.1 × 10^5^	30 hours 7, 16, and 23 days
Terrovitis et al., 2008 [58]	^124^I ^99m^Tc	Immediately	Wistar Kyoto rats	Rat CDCs	Intravenous	2 × 10^6^	1 hour
Lee et al., 2011 [59]	^18^F-FHBG	30 minutes	Dogs	Canine iPSCs	Intramyocardial	1 × 10^6^	8 hours
Liu et al., 2012 [60]	^18^F-FHBG	Immediately	SCID Beige mice	Human CDCs	Intramyocardial	1 × 10^6^	1, 7, 14, 21, and 28 days
Templin et al., 2012 [62]	^123^I	Not specified	Pigs	Human iPSCs	Intramyocardial	1 × 10^8^	1 and 5 days and 12-15 weeks
Lan et al., 2012 [61]	^18^F-FHBG	Immediately	SCID Beige mice	Human CDCs	Intramyocardial	1 × 10^6^	1, 7, 14, 21, and 28 days
Yan et al., 2013 [63]	^18^F-FHBG	10 minutes	Nude mice	Murine BM-MSCs	Intramyocardial	1 × 10^6^	Same day, 3 and 7 days
Pei et al., 2014 [64]	^18^F-FHBG	Immediately	Sprague-Dawley rats	Rat BM-MSCs	Intramyocardial	5 × 10^6^	2, 3, and 7 days
Lee et al., 2015 [65]	^99m^Tc	7 days	Dogs	Canine ADSCs	Intramyocardial	1 × 10^7^	2 hours and 1, 2, 5, 7, 9, and 12 days

^123^I: iodine-123; ^124^I: iodine-124; ^18^F: fluorine-18; ^99m^Tc: technetium-99m; ADSCs: adipose tissue-derived stem cells; BM-MSCs: bone marrow mesenchymal stem cells; CDCs: cardiac-derived stem/progenitor cells; FHBG: 9-[4-fluoro-3-(hydroxymethyl)butyl]guanine derivatives; iPSCs: induced pluripotent stem cells; SCID: severe combined immunodeficiency.

**Table 3:** Clinical studies that used direct radiopharmaceutical labeling for cell therapies in cardiology.

Study reference	Radiopharmaceutical	Type of lesion	Cell type	Route	Number of patients	Number of cells injected	Imaging time points
Hofmann et al., 2005 [72]	^18^F-FDG	Acute myocardial infarction	BM-MNCs	Intracoronary Intravenous	12	Not specified	75 min
Blocklet et al., 2006 [71]	^18^F-FDG ^111^In-oxine	Acute myocardial infarction	PB-MNCs	Intracoronary	6	2–4 × 10^6^	1 hour (^18^F-FDG) 19 and 43 hours (^111^In-oxine)
Kang et al., 2006 [73]	^18^F-FDG	Acute or nonacute myocardial infarction	PB-MNCs	Intracoronary Intravenous	20	Not specified	2, 4, and 20 hours
Goussetis et al., 2006 [74]	^99m^Tc-HMPAO	Chronic ischemic cardiomyopathy	BM-MNCs	Intracoronary	8	0.8 × 10^7^	1 and 24 hours
Caveliers et al., 2007 [66]	^111^In-oxine	Chronic ischemic cardiomyopathy	PB-MNCs	Intracoronary	8	5–35 × 10^6^	2 and 12 hours
Penicka et al., 2007 [75]	^99m^Tc-HMPAO	Acute or nonacute myocardial infarction	BM-MNCs	Intracoronary	10	24.2–57.0 × 10^8^	2 and 20 hours
Kurpisz et al., 2007 [67]	^111^In-oxine	Acute myocardial infarction	BM-MNCs	Intracoronary	3	2–4 × 10^6^	24 hours
Schots et al., 2007 [68]	^111^In-oxine	Nonacute myocardial infarction	PB-MNCs	Intracoronary	8	5–10 × 10^6^ 18–50 × 10^6^	2 and 12 hours
Schächinger et al., 2008 [69]	^111^In-oxine	Acute or nonacute myocardial infarction	PB-MNCs	Intracoronary	20	15 × 10^6^	1 and 24 hours
Dedobbeleer et al., 2009 [70]	^18^F-FDG	Nonacute myocardial infarction	PB-MNCs	Intracoronary	7	1.8 × 10^7^	1 hour
Silva et al., 2009 [76]; Moreira et al., 2011 [77]	^99m^Tc-HMPAO	Acute myocardial infarction	BM-MNCs	Intracoronary IRCV	30	1 × 10^7^	4 and 24 hours
Musialek et al., 2011 [78]	^99m^Tc-HMPAO	Acute myocardial infarction	BM-MNCs	Intracoronary	34	0.92–7.54 × 10^6^	1 hour
Barbosa da Fonseca et al., 2011 [80]	^99m^Tc	Chronic chagasic cardiomyopathy	BM-MNCs	Intracoronary	6	4.4 × 10^8^	1, 3, and 24 hours
Kollaros et al., 2012 [81]	^99m^Tc-HMPAO	Chronic myocardial infarction	BM-MNCs	Intracoronary	13	Not specified	1 hour
Musialek et al., 2013 [79]	^99m^Tc-HMPAO	Recent myocardial infarction	BM-MNCs	Intracoronary	31	4.3 × 10^6^	1 hour
Vrtovec et al., 2013 [82]	^99m^Tc-HMPAO	Nonischemic dilated cardiomyopathy	BM-MNCs	Intracoronary transendocardial	40	1 × 10^6^	18 hours
Haddad et al., 2015 [83]	^99m^Tc-HMPAO	Nonischemic dilated cardiomyopathy	PB-MNCs	Transendocardial	37	75 × 10^6^	2 and 18 hours

^111^In: indium-111; ^18^F: fluorine-18; ^99m^Tc: technetium-99m; BM-MNCs: bone marrow mononuclear cells; FDG: fluorodeoxyglucose; HMPAO: hexamethylpropyleneamine oxime; IRCV: interstitial retrograde coronary venous; PB-MNCs: peripheral blood mononuclear cells.
